# Seeing through multimode fibers using real-valued intensity transmission matrix with deep learning

**DOI:** 10.1364/OE.553949

**Published:** 2025-04-03

**Authors:** Ziyu Ye, Tianrui Zhao, Wenfeng Xia

**Affiliations:** School of Biomedical Engineering and Imaging Sciences, King’s College London, 4th Floor, Lambeth Wing St Thomas’ Hospital, London SE1 7EH, United Kingdom

## Abstract

Multimode fibers (MMFs) are emerging as a highly attractive technology for applications in biomedical endoscopy and telecommunications, thanks to their ability to transmit images and data through a large number of transverse optical modes. However, light transmission through MMFs suffers from distortions caused by mode dispersion and coupling. While recent deep learning advancements have shown potential for improving image transmission through MMFs, these methods typically require an extensive training dataset and often exhibit limited generalization capability. In this work, we propose a hybrid approach that combines a real-valued intensity transmission matrix (RVITM) with deep learning for enhanced image retrieval through MMFs. Our approach first characterizes the MMF and retrieves images using a RVITM algorithm, followed by refinement with a hierarchical, parallel multi-scale (HPM)-attention U-Net to improve image quality. Experimental results demonstrated that our approach achieved high-quality reconstructions, with structural similarity index (SSIM) and peak signal-to-noise ratio (PSNR) values of up to 0.9524 and 33.244 dB, respectively. This approach also offers strong generalization capabilities, requires fewer training samples and converges more quickly compared to purely deep learning-based methods reported in the literature. These results highlight the potential of our method for ultrathin endoscopy applications and spatial-mode multiplexing in telecommunications using MMFs.

## 1. Introduction

MMFs have demonstrated significant potential to revolutionise application in ultrathin endoscopy and telecommunications. [1]. For endoscopy, coherent fiber bundles (CFBs) are commonly used and each core in the bundle corresponds to an image pixel, however MMFs offer a larger number of transverse optical modes. This capability allows MMFs to achieve 1-2 orders of magnitude higher spatial resolution while simultaneously reducing costs by 2-3 orders of magnitude [2]. Further, MMF-based endoscopes provide greater flexibility as the focal spot diameter, shape and focal plane can be adjusted in contrast to CFB-based implementations [3]. For telecommunications, the larger number of modes in MMFs could also be useful for mode group diversity multiplexing data transmission [4]. However, the light transmission through MMFs is significantly affected by mode dispersion and coupling, resulting in random-like speckle patterns with chaotic intensity information [,^5^–^7^].

Over the past decade, several research groups [8–11] have investigated and demonstrated transmission matrix (TM)-based methods that can characterise the information of light field through complex media, such as MMFs [12]. TM is defined as a matrix with transmission constants that include amplitude, phase information or both, and that can be used to represent the linear relationship between the input and output light fields. However, these matrices, commonly known as complex transmission matrices (TMs), have several limitations. They are sensitive to noise and require precise phase information. This sensitivity can lead to significant errors in the reconstructed images or in the light focusing through MMFs. Noise can cause by various sources, such as mechanical vibrations, fluctuations in light sources. The requirement for precise phase information increases the cost and complexity of endoscope imaging systems and also degrades their stability. Devices like liquid crystal spatial light modulator (SLM) are commonly used to provide precise phase control but tend to operate at slower speeds [2,3,5,6]. To address these challenges, the authors group have introduced the real-valued intensity transmission matrix (RVITM) method, which uses a digital micromirror device (DMD) that offers a much faster modulation speed with amplitude modulation [13]. This method simplifies the characterization process by finding the approximate linear relationship between the input and output light intensity distributions and allows for high-speed imaging without the need for phase information. The use of a high-speed DMD significantly enhances the speed of wavefront shaping by several orders of magnitude, enabling real-time imaging by raster-scanning of a focal beam at the distal end of an MMF [14–16], and offering promising capabilities for high-speed focusing through biological tissue [17].

Recent research has increasingly focused on leveraging deep learning methods to directly learn the non-linear mapping relationship between input and output light fields transmitted through MMFs [18–23]. Ju et al., developed a dual-function MMF imaging system using a fully connected encoder-decoder architecture for image reconstruction [20]. Zhu et al., proposed a simple neural network architecture that achieved high-fidelity images through a MMF [25]. These learning-based methods effectively reconstruct input images from the output light filed, which undergone complex distortions caused by mode dispersion and coupling in MMFs. However, traditional deep learning methods [19–23] have serval limitations. A notable challenge is the requirement to acquire extensive dataset containing more than twenty thousand images, each sized 192 × 192 or bigger, can result in training times exceeding one day. Furthermore, these models usually have high demand on computational resources, such as graphic performance units (GPUs). Notably, their performance can be heavily relied on the quality of the training data and the generalisation ability of these models are usually suboptimum. Therefore, there is a strong need for alternative approaches that can harness the strengths of deep learning while mitigating the extensive data requirements, thereby enhancing their practicality for applications with limited datasets and improving their generalisation capabilities.

In this work, we propose a hybrid approach that combines deep learning and RVITM to enhance image retrievals though MMFs. By combining the benefits of RVITM's incorporation of physical prior knowledge with deep learning's capacity to capture nonlinear relationships, we aim to enhance image quality and retrieval speed while minimising the requirement for extensive training data. Our approach leverages RVITM to supply physical priors for image reconstruction, thereby reducing the complexity that the deep learning model needs to handle. This not only accelerates the training process but also improves the generalisation capability of the model. Furthermore, we evaluate our method's ability to enhance the fidelity of reconstructed images while maintaining high computational efficiency. This combination of physical modelling and deep learning provides a promising approach for improving MMF-based imaging systems and achieving high-quality image retrievals in biomedical endoscopy and telecommunication applications.

## 2. Methods and materials

### 2.1. Real-valued intensity transmission matrix-assisted learning

**Fig. 1.**
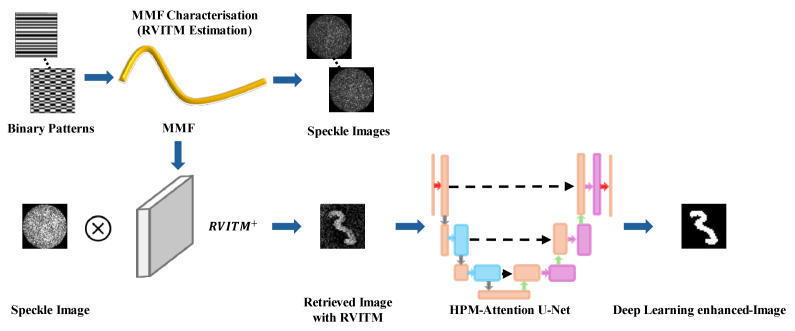
Schematic diagram of image retrieval via MMF using real-valued intensity of transmission matrix-assisted learning. In the characterization step, a set of input binary Hadamard patterns are projected onto the proximal end of a multimode fiber. The speckle images captured by the camera at the distal fiber end, are used to calculate RVITM. Image retrieval is then conducted using the pseudo-inverse of RVITM, which is subsequently used as the input of the HPM-Attention-U-Net to enhance the quality of the final reconstructed image.

The concept of RVITM-assisted learning for image retrieval through MMFs is illustrated in Fig. 1. This approach consists of two steps: first, image retrieval is performed using RVITM algorithm described in Ref. [13]; and second, the retrieved images are further processed to enhance image fidelity by training a neural network.

In the first step, RVITM is first measured for a MMF using a set of binary DMD patterns as input, and image reconstruction is performed based on linear inversions to retrieve an input DMD pattern. Further details about the MMF characterization and image retrieval are provided in Section 2.2.

The second stage involves training HPM-Attention U-Net model which is then used to reconstruct images based on the retrieved speckle images in the first step. Although recent optical fiber imaging scheme in deep learning is to directly learn the mapping relationship between speckle images and original images [18], these modes often require number of training images and time-consuming iterative processes, limiting their practical applicability. In contrast, the mapping between the retrieved speckle images and the original images is simpler, reducing the need for extensive training data and shortening the training time [26]. Details of the network architecture and experiments are descried in Section 2.3 and 2.4, respectively.

### 2.2. MMF characterization and image retrieval based on RVITM

In the MMF characterization process, a binary Hadamard matrix *H* was employed and displayed on a DMD. The Hadamard matrix was a square matrix with the size of N, and with elements of either -1 or +1. However, since a DMD provided binary modulation with values of ‘0’ and ‘1’, the Hadamard matrix was converted into two binary matrices $H_{1}$ and $H_{2}$ , using the formulas $H_{1}=\frac{H+1}{2}$ and $H_{2}=\frac{-H+1}{2}$ separately. This process resulted in a total of 2N binary patterns as the MMF input, where each matrix was squared and contained N pixels. The RVITM thus connects the output speckle and input binary pattern as follows based on the pseudo-linear relationship reported in Ref. [13]:

(1) $$\left[\begin{array}{ccc} I_{1}^{1} & \cdots & I_{1}^{j}\\ \vdots & \ddots & \vdots \\ I_{i}^{1} & \cdots & I_{i}^{j} \end{array}\right]=RVITM\cdot [H_{1},H_{2}],$$

(2) $$\left[\begin{array}{ccc} 2I_{1}^{1}-I_{1}^{1} & \cdots & 2I_{1}^{j}-I_{1}^{1}\\ \vdots & \ddots & \vdots \\ 2I_{i}^{1}-I_{i}^{1} & \cdots & 2I_{i}^{j}-I_{i}^{1} \end{array}\right]=RVITM\cdot [2H_{1}-1,2H_{2}-1],$$

where *I* represents the intensity distribution of the output speckle, *i* is the $i^{th}$ pixel of the output speckle, *j* is the $j^{th}$ number of the output speckle” Given the Hadamard matrix properties, all the values in the first binary pattern were ‘1’. $\left[2H_{1}-1,2H_{2}-1\right]$ on the right-hand side was transformed to [H,−H] . Due to the Hadamard matrix properties again, ${[H,-H]}^{T}$ equals ${[H,-H]}^{-1}$ . Therefore, the RVITM can be further expressed by multiplying ${[H,-H]}^{T}$ at both sides of Eq. (2):

(3) $$RVITM=\left[\begin{array}{ccc} 2I_{1}^{1}-I_{1}^{1} & \cdots & 2I_{1}^{j}-I_{1}^{1}\\ \vdots & \ddots & \vdots \\ 2I_{i}^{1}-I_{i}^{1} & \cdots & 2I_{i}^{j}-I_{i}^{1} \end{array}\right]\cdot {[H,-H]}^{T},$$

After calculation, the RVITM value was obtained by correlating the intensity of the output speckles with the input binary patterns using simple matrix manipulation. The image retrieval process involved using the obtained RVITM along with the output speckles captured by the camera to reconstruct the input image. The reconstruction follows the equation below:

(4) $$I_{re}=RVITM^{+}\cdot I_{sp},$$

where $I_{re}$ is the retrieved image and $I_{sp}$ is the corresponding speckle image. $RVITM^{+}$ represents the pseudo-inverse of RVITM [13].

### 2.3. Network architecture

**Fig. 2.**
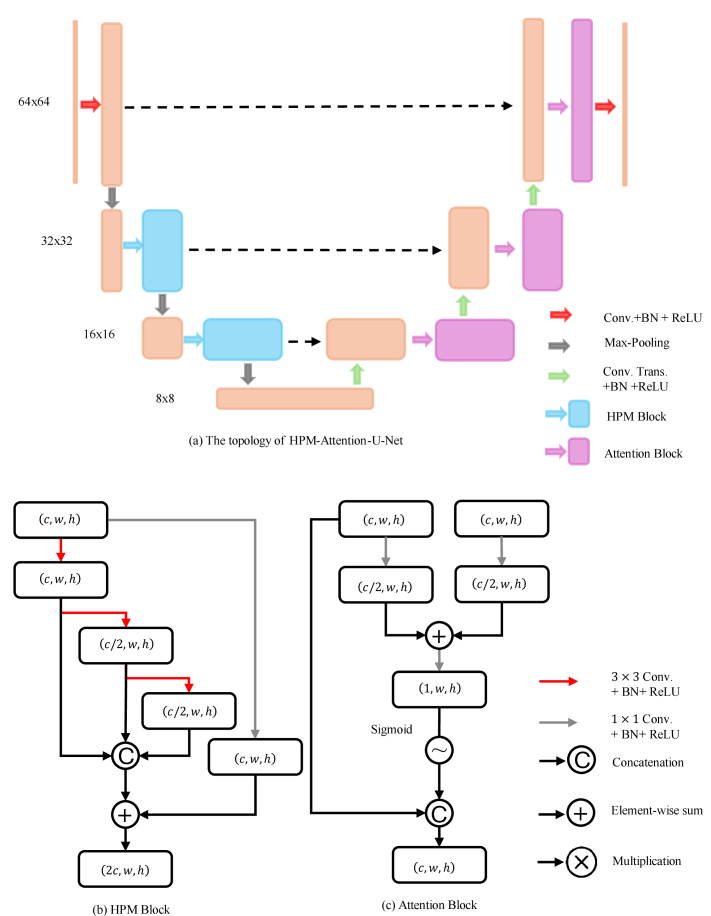
The structure of the HPM-Attention U-Net. (a) the topology of the HPM-Attention-U-Net is symmetric with three steps of pooling. At each down-sampling stage, a HPM block is employed to process incoming data, followed by the placement of an attention block at each corresponding up-sampling stage. (b) a HPM block can increase the receptive field size without increasing the number of parameters. Additionally, there is an additional 1 × 1 convolution step to increase the number of channels of the output feature map that can be fed to the subsequent layer. (c) an attention block has two kinds of input including the feature map from the preceding up-sampling step and the feature map of the current HPM block at the current resolution via the skip connection.

The structure of the proposed HPM-Attention-U-Net is illustrated in Fig. 2(a). This network was designed to enhance the quality and fidelity of the images retrieved using the RVITM. The enhancement was achieved by integrating the standard U-Net [27] with hierarchical, parallel multi-scale blocks (see Fig. 2(b)) [28] and attention blocks (see Fig. 2(c)) [29]. These additions enabled the model to effectively capture both local and global features at various resolutions without significantly increasing the number of model parameters. The use of skip connections helped preserve spatial information across different resolutions, while the attention mechanism implicitly suppressed irrelevant background features, eliminating the need for explicit steps, such as cropping the region of interest (ROI) in a two-stage network approach. In addition, by removing one pooling step, the network maintained a starting resolution of 64 × 64 pixels, reducing the computational complexity and model size.

### 2.4. Experimental setup and training details

**Fig. 3.**
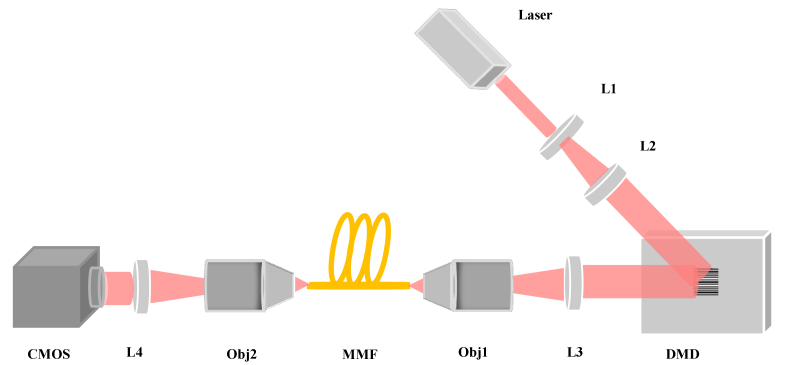
Schematic diagram of the RVITM experiment set up. L1-L4: tube lens; Obj1 and Obj2: microscope objectives; DMD is a digital micromirror device; MMF: multimode fiber, CMOS: camera.

The experimental setup for the RVITM characterization and the speckle images collection is shown in Fig. 3. After collimation and beam expansion, the light beam emitted from a pulsed laser (532 nm, 2 ns, SPOT-10-200-532, Elforlight) was spatially modulated by a DMD (DLP7000, 768 × 1080 pixels, Texas Instruments) using an input set of binary patterns. The DMD plane was then imaged on to the proximal end of an MMF (200 μm-core, NA = 0.22, length = 1 m, M122L01, Thorlabs) through a tube lens (AC254-050A-ML, focal length = 50 mm, Thorlabs) and an objective (20×, NA = 0.4, RMS20X, Thorlabs). Finally, the output speckle images, from the fiber’s distal end, corresponding to each input pattern, were captured by a CMOS camera (C11440-22CU01, Hamamatsu Photonics) magnified by an objective (20×, NA = 0.4, RMS20X, Thorlabs) and a tube lens (AC254-0100-A-ML, focal length = 100 mm, Thorlabs).

Each micromirror of the DMD was set as a single input image pixel to switch the ‘ON’ or the ‘OFF’ pixel. The input binary pattern consisted of 64×64(N=4096) pixels, while the pixels count of the output speckle pattern consisted of 360×360(M=129,600) . Using the Eq. (1) and the set of 8192 binary patterns as the input, the RVITM characterization step was performed, producing an RVITM of size M×N .

Table 1 outlines the datasets used in the experiment: the D1 dataset, consisting of 2,048 images from the modified National Institute of Standards and Technology (MNIST) dataset [30]; the D2 dataset, comprising D1 along with an additional 2,022 images of 13 types of complex targets; and the D3 dataset, a test set containing 24 images featuring a cat in various poses, used to assess the system's robustness and the generalization capability. These images, resized to 64×64 pixels to meet the input DMD pattern requirements, were utilised to generate speckle images based on the experiment set up described above. Subsequently, the retrieved images were reconstructed using the pseudo-inverse of RVITM, as detailed in Eq. (2).

**Table 1.** Details of different datasets

Name	Sizes	Label
D1	2048	Handwritten digits
D2	2048 + 2022	Handwritten digits with different complex target
D3	24	Cat

To further evaluate the performance of the proposed network, several previously established deep learning models were investigated, including the full-connected auto-encoder (FC-AE) [24], convolutional auto-encoder (CNN-AE) [19], convolutional auto-encoder with self-normalizing neural network (CNN-AE-SNN) [20], U-Net [27], and Attention-U-Net [29]. All models were implemented and trained using the retrieved images from the D1 and D2 datasets on an NVIDIA GeForce RTX 2080 Ti GPU with 11 GB of memory. The training was conducted using PyTorch 1.9.1 with the Adam optimizer, an initial learning rate of $1.0\times 10^{-4}$ , a batch size of 64, and 90 epochs to minimize the mean square error. The D1 and D2 datasets were split into training, validation, and test sets with a ratio of 7:1:2, respectively. The best model parameters were saved when the validation loss reached its minimum.

### 2.5. Performance metrics

First, to assess image quality and fidelity, we employed structural similarity index (SSIM) and peak signal-to-noise ratio (PSNR) as the performance metrics. SSIM is a method for measuring the similarity of two images to quantify the fidelity between the reconstructed image and the target image, considering the changes in texture, illumination, and contrast. The SSIM between two images *x* and *y* is given by:

(5) $$SSIM(x,y)=\frac{\left(2\mu_{x}\mu_{y}+C_{1})\cdot (2\sigma_{xy}+C_{2}\right)}{\left(\mu_{x}^{2}+\mu_{y}^{2}+C_{1})\cdot (\sigma_{x}^{2}+\sigma_{y}^{2}+C_{2}\right)},$$

where $\mu_{x}$ and $\mu_{y}$ are the mean intensities of the entire images *x* and *y* respectively, $\sigma_{x}^{2}$ and $\sigma_{y}^{2}$ denote their variance, $\sigma_{xy}$ indicates the covariance of the images and $C_{1}$ and $C_{2}$ are constants to stabilise the division with a weak denominator. PSNR is a metric that is commonly used to evaluate the quality of the reconstructed image by measuring the peak error. Here, the signal represents the target image, and the noise is the error introduced by reconstruction process. A higher PSNR indicates that the reconstructed image is of a higher quality. PSNR is defined as:

(6) $$PSNR=10\times \log_{10}\left[\frac{{\left(2^{N}-1\right)}^{2}}{\frac{1}{mn}\sum_{i=1}^{m}\sum_{j=1}^{n}{\left(I_{x}-I_{y}\right)}^{2}}\right],$$

where $I_{x}$ and $I_{y}$ are the pixel intensities of the target and the reconstructed images, respectively, *m* and *n* represent the height and width of the image, and *N* is the number of bits per pixel.

## 3. Results

**Fig. 4.**
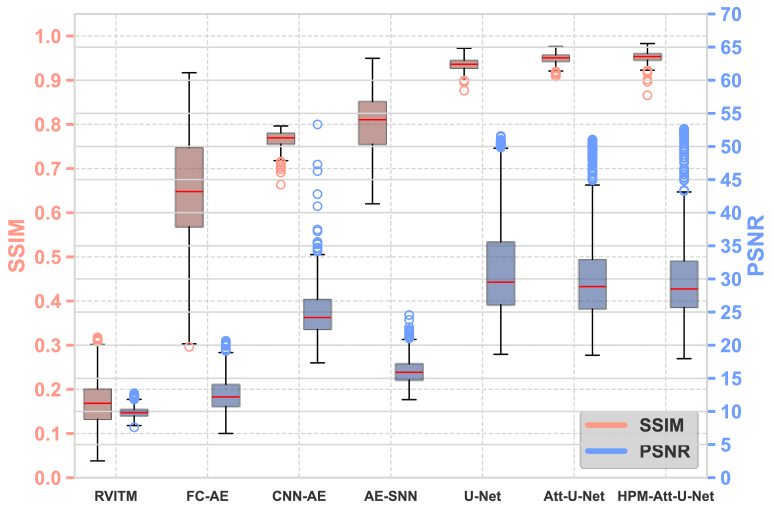
Box-and-whisker plots comparing the performance of various models on the D1 test set. The left y-axis represents SSIM values (orange), while the right y-axis shows PSNR values in dB (blue). Each box plot displays the interquartile range (IQR), with the upper and lower edges corresponding to the 75th and 25th percentiles, respectively, and the red line inside the box indicating the median. The whiskers extend from the edges of the box to the maximum and minimum values within 1.5 times the IQR, while any data points outside this range are considered outliers.

**Fig. 5.**
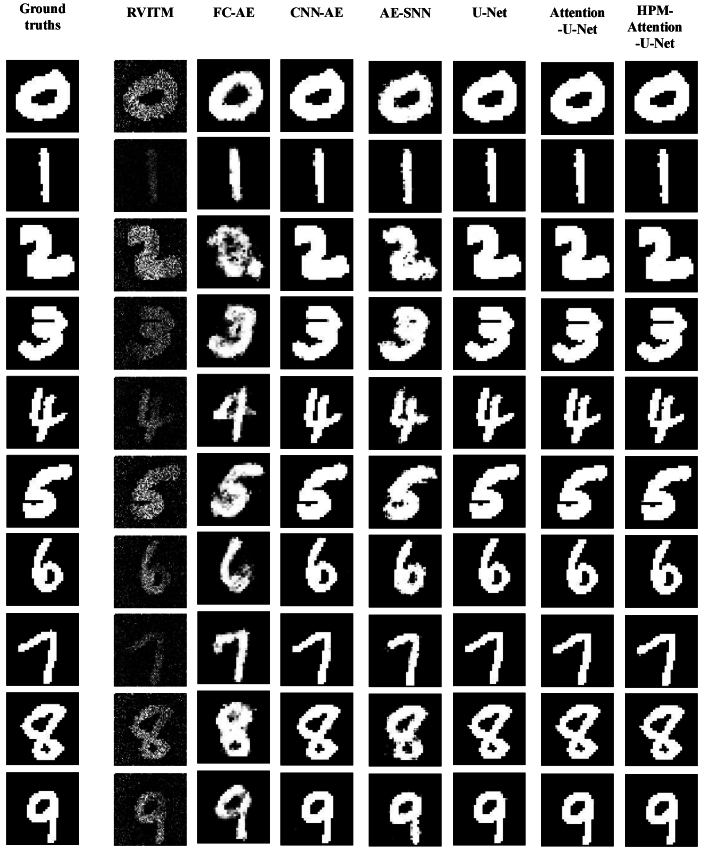
Examples of reconstructed images with handwritten digits using RVITM in combination with different deep learning-based models. The first column is the target images, the second column is the corresponding retrieved images using RIVTM alone, and the rest of the columns are the enhanced images by proposed hybrid models combing RVITM with different deep learning-based models.

The performance of different models, along with the examples of reconstructed images on the D1 test set, is shown in Figs. 4 and 5. HPM-Attention U-Net achieved the highest average values of SSIM and PSNR on the D1 test set, at 0.9524 and 33.244 dB, respectively, indicating its strong capability to learn the mapping between target and retrieved images that incorporate prior knowledge of the physical model and numerical reconstruction. The box plot in Fig. 4 shows the distribution of the SSIM and PSNR values across all models. In general, all the deep learning models under investigation had substantial improvements compared to RVITM, in terms of SSIM and PSNR. The HPM-Attention U-Net had the highest median SSIM values, and the HPM-Attention U-Net, U-Net and Attention U-Net, exhibited the highest PSNR values across all the models. Moreover, HPM-Attention U-Net converged more rapidly, requiring only 30 epochs and a total training time of approximately 508 seconds. This demonstrated its efficiency and accuracy, even with a small-scale dataset. Additionally, as shown in Figs. 4 and 5, both the performance metrics and reconstructed images indicate that U-Net-based algorithms perform better than AE-based algorithms, with the latter producing more distorted images when trained on small datasets. This distortion is likely attributed to small-scale datasets that provided a limited sample space, reducing the effectiveness of AE-based algorithms.

**Fig. 6.**
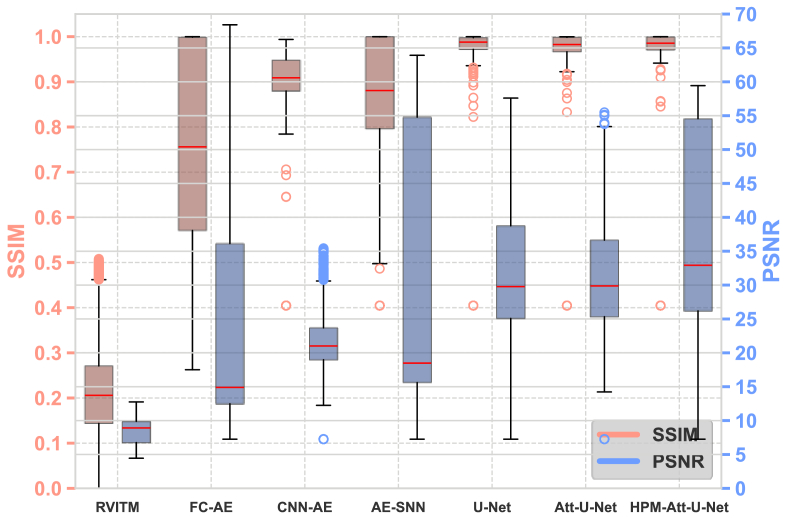
Box-and-whisker plots comparing the performance of various models on the D2 test set. The left y-axis represents SSIM values (orange), while the right y-axis shows PSNR values in dB (blue). Each box plot displays the interquartile range (IQR), with the upper and lower edges corresponding to the 75th and 25th percentiles, respectively, and the red line inside the box indicating the median. The whiskers extend from the edges of the box to the maximum and minimum values within 1.5 times the IQR, while any data points outside this range are considered outliers.

**Fig. 7.**
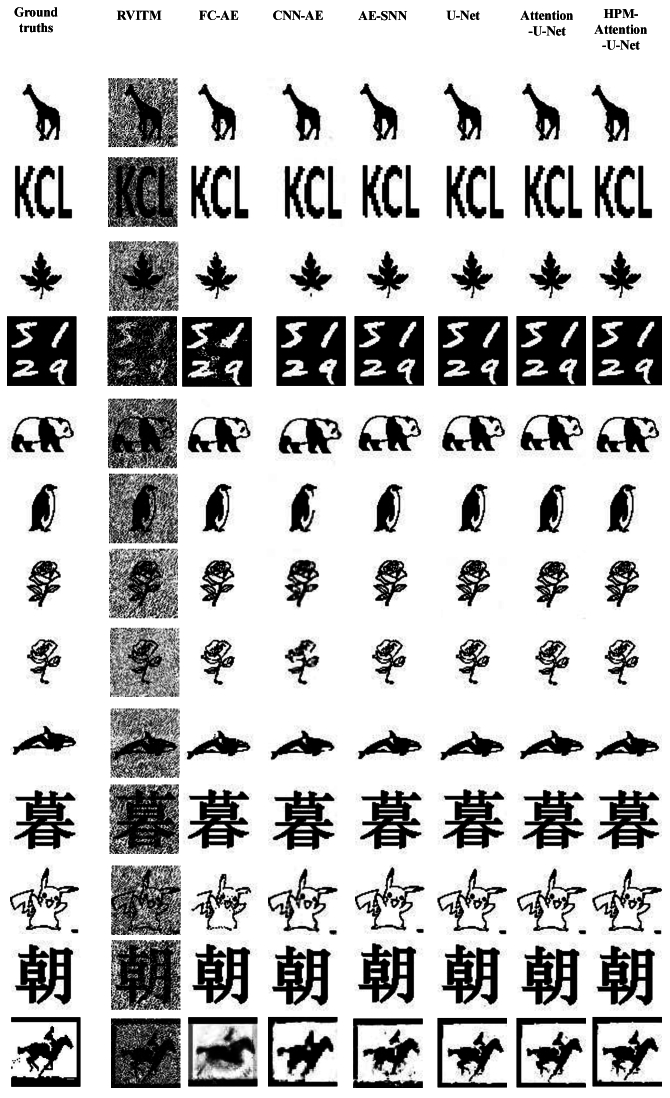
Examples of reconstructed images with 13 types of complex targets using RVITM in combination with different deep learning-based models. The first column is the target images, the second column is the corresponding retrieved images using RIVTM alone, and the rest of the columns are the enhanced images by proposed hybrid models combing RVITM with different deep learning-based models.

The performance of different deep learning models, along with the examples of reconstructed images on the D2 test set are shown in Figs. 6 and 7, respectively. Similarly, all the deep learning models outperformed the original RVITM approach with a large margin. The HPM-Attention U-Net achieved the highest average SSIM and PSNR values on the D2 test set, at 0.9330 and 37.3186 dB, respectively. However, the overall SSIM values across all models are lower on the D2 test set compared to the D1 test set, primarily due to limitations in the dataset, because only 70 images per complex target were used for training, and the “horse” category contains only 14 images. Furthermore, as shown in Fig. 6, some SSIM values are close to 1, and PSNR values illustrate a wide variability across all deep learning-based models. This variability is likely due to the use of only 12 types of static complex targets with data augmentation, which included rotation, translation, and scaling. Deep learning-based method could effectively learn the mapping relationship between the targets and the retrieved images. When the differences in the data were not large, U-Net-based models exhibited similar performance. However, the HPM-Attention U-Net had fewer lower outlier points, indicating more consistent performance and better robustness in handling variations. Additionally, the training time for the HPM-Attention U-Net increased to approximately 1,015 seconds due to the larger size of the training set.

**Fig. 8.**
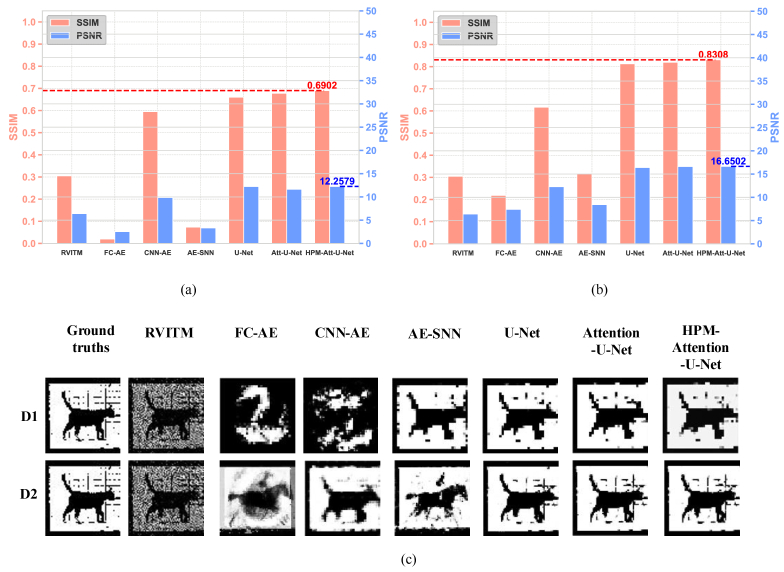
Comparison of model performance on the D3 dataset. (a) Reconstruction accuracy measured using RVITM and various deep learning-based models trained on the D1 dataset. (b) Reconstruction accuracy measured using RVITM and deep learning-based models trained on the D2 dataset. (c) Examples of reconstructed images: the first row shows results from models trained on the D1 dataset, and the second row displays results from models trained on the D2 dataset.

To demonstrate the generalisation ability of the HPM-Attention-U-Net on unseen data, further evaluation was test on the D3 dataset, as shown in Fig. 8. When using the trained model on the D1 dataset, the HPM-Attention U-Net achieved the highest average SSIM and PSNR values among all models, at 0.6902 and 12.2579 dB, respectively. With additional training images containing different complex targets from the D2 dataset, HPM-Attention U-Net achieved even higher average SSIM and PSNR values of 0.8308 and 16.6502, respectively. By updating the retrieved images, the trained HPM-Attention U-Net could enhance the quality of the retrieved image more accurately without requiring time-consuming retraining.

## 4. Discussion

In this work, we proposed a deep learning-based method that utilise the physical prior knowledge from the RVITM to enhance image retrieval through MMFs. The network architectures shown in Fig. 4 and Fig. 6 were trained using images retrieved with RVITM. The proposed HPM-Attention U-Net model was then compared with previously reported deep learning-based image retrieval approaches, as detailed in Table 2. The pre-trained AE, AE-SNN, Dense-U-Net, FC-encoder-decoder, and U-Net models were trained directly using speckle patterns. In contrast, the AM-U-Net, MU-DLN, and TM-U-Net models utilized speckle patterns augmented with different physical priors for training. Specifically, AM-U-Net implemented a complex dual-polarization-based data acquisition system to capture two types of polarized speckle images, including P-polarization and S-polarization, MU-DLN incorporated a pre-physical step to collect linear-polarization images as its dataset, and TM-U-Net employed optimized patterns that were reconstructed using a calibrated TM. Although AM-U-Net and TM-U-Net required fewer training images, their training times were longer, and their performance was worse. The proposed HPM-Attention U-Net enhances the standard U-Net and Attention U-Net architectures. Its Hierarchical, Parallel Multi-Scale (HPM) module improves feature extraction at different scales during down-sampling, while attention blocks in the up-sampling stages suppress irrelevant background features without significantly increasing model complexity. Moreover, alternative methods rely on more complex data acquisition systems, reducing overall system stability. By incorporating RVITM as a physical prior, our approach provides a high-speed, reference-arm-free solution that minimizes characterization and data collection time, offering a more efficient and stable alternative to existing methods. In contrast, the RVITM method employed in our work acts as physical prior knowledge, providing a high-speed, reference-arm-free solution that requires minimal time for characterization and data collection.

**Table 2.** Comparison of image reconstruction performance between previously reported networks and our hybrid method that combines deep-learning and RVITM.

Neural Network	Training set (pairs)	Training Time (s)	SSIM	PSNR (dB)
Pre-trained AE [19]	3,500	Unknown	0.6513	17.7
AE-SNN [24]	4,000	600	0.6996	Unknown
AM-U-Net [29]	300	3894.39	0.8772	31.83
Dense-U-Net [19]	11,000	54000	0.7900	36.44
FC-encoder-decoder [24]	27,000	8421	0.8660	22.13
MU-DLN [20]	5,000	Unknown	0.8040	22.29
U-Net [26]	18,000	54000	0.7159	24.35
TM-U-Net [26]	600	1140	0.8897	33.29
HPM-Attention-U-Net (**Ours**)	2048	**508**	**0**.**9524**	33.24

Unlike traditional deep learning methods [18–23], which typically require large datasets, our model achieves faster convergence with fewer training images. The combination of RVITM and HPM-Attention U-Net allows for improved efficiency and accuracy in image retrieval. As noted in Ref. [13], the computation time for RVITM estimation increased with both the input and output pixel counts. For 4096-pixel images, the RVITM estimation process took ∼21.04 ms, including ∼0.04 ms for data acquisition using DMD operating at ∼23 kHz, ∼21 ms for transferring data back to the PC and RVITM calculation for image retrieval. The inference time of HPM-Attention U-Net was 5 ms, resulting in a total processing time of 26.04 ms using a desktop PC (Intel i9 9900 k, 3.6 GHz with RTX 2080Ti). Since actual image retrieval is determined by the inference time (5 ms), our method is expected to achieve a sufficient frame rate for most biomedical endoscopy applications. However, for telecommunications, this speed remains too slow for many practical scenarios.

Given that RVITM approximates a linear relationship, the image retrieval accuracy of our method may be lower compared to traditional transmission matrix (TM) methods, which incorporates phase information for higher precision [13]. However, several strategies can be employed to enhance the results. For instance, optimising the network architecture by introducing dense connections can enhance feature extraction and preserve feature information at the different scales [31]. Additionally, strategies such as fine-tuning and using pre-trained models can accelerate convergence and improve accuracy [19]. Furthermore, incorporating physical rules into the model can boost its learning and generalization capabilities [23].

This study demonstrates the capability of retrieving binary images due to the digital micromirror device (DMD) used, which allows only binary amplitude modulations. Future research will explore the use of liquid crystal spatial light modulators as applied in previous studies [18,32] or advanced DMD-based techniques [33,34] that enable grayscale amplitude modulation to assess our method's effectiveness in grayscale image retrieval. As illustrated in Fig. 8, the current deep learning model has some limitations in handling the unseen data for image retrieval. We acknowledge that they are based on a limited dataset that might not fully represent other types of data. In future work, we aim to conduct extensive experiments using diverse datasets that encompass different modalities and object complexities. This will allow us to more comprehensively assess the general applicability and effectiveness of our proposed method across various scenarios. Addressing the challenge posed by ambient disturbances, including temperature fluctuations, fiber bending, and other environmental factors, remains a crucial area for further research. We are considering the integration of deep learning algorithms and prior physical knowledge to construct a different database containing speckle images captured under various fiber bending condition. Such a database could be used to classify different fiber configurations and enhance image retrieval accuracy for the real-word applications in biomedical endoscopy and telecommunications [35].

## 5. Conclusion

In conclusion, we developed a method that significantly improves the quality and fidelity of images retrieved from MMF using an RVITM-based approach assisted with an HPM-Attention U-Net model. The RVITM simplifies the characterization process by using intensity-only information, enabling fast image retrieval. By leveraging the strengths of deep learning, our system achieves high-quality reconstruction with fewer training samples and faster convergence times.

## Data Availability

Data underlying the results presented in this paper are available at [36].

## References

[1] Psaltis D. Moser C., “Imaging with multimode fibers,” Optics and Photonics News 27(1), 24–31 (2016). 10.1364/OPN.27.1.000024

[2] Plöschner M. Tyc T. Čižmár T., “Seeing through chaos in multimode fibers,” Nat. Photonics 9(8), 529–535 (2015). 10.1038/nphoton.2015.112

[3] Andresen E. R. Sivankutty S. Tsvirkun V. et al., “Ultrathin endoscopes based on multicore fibers and adaptive optics: a status review and perspectives,” J. Biomed. Opt. 21(12), 121506 (2016). 10.1117/1.JBO.21.12.121506 27722748

[4] He Z. Fan Y. Ye F. et al., “An optical MMF communication system based on mode group diversity multiplexing and Space Time coding,” Asia Communications and Photonics Conference and Exhibition, Shanghai, China, pp. 667–668, (2010)

[5] Hui R. Mosk S. Rotter S., “Shaping the propagation of light in complex media,” Nature Physics 18(1), 997–1007 (2022). 10.1038/s41567-022-01677-x

[6] Di Leonardo R. Bianchi S., “Hologram transmission through multi-mode optical fibers,” Opt. Express 19(1), 247–254 (2011). 10.1364/OE.19.000247 21263563

[7] Gigan S. Katz O. de Aguiar H. B.. et al., “Roadmap on wavefront shaping and deep imaging in complex media,” J. Phys.: Photonics 4(4), 042501 (2022). 10.1088/2515-7647/ac76f9

[8] Čižmár T. Dholakia K., “Shaping the light transmission through a multimode optical fiber: complex transformation analysis and applications in biophotonics,” Opt. Express 19(20), 18871–18884 (2011). 10.1364/OE.19.018871 21996829

[9] Cižmár T. Dholakia K., “Exploiting multimode waveguides for pure fiber-based imaging,” Nat. Commun. 3(1), 1027 (2012). 10.1038/ncomms2024 22929784 PMC3432471

[10] Boniface A. Blochet B. Dong J. et al., “Noninvasive light focusing in scattering media using speckle variance optimization,” Optica 6(11), 1381–1385 (2019). 10.1364/OPTICA.6.001381

[11] Boniface A. Mounaix M. Blochet B. et al., “Transmission-matrix-based point-spread-function engineering through a complex medium,” Optica 4(1), 54–59 (2017). 10.1364/OPTICA.4.000054

[12] Rotter S. Gigan S., “Light fields in complex media: mesoscopic scattering meets wave control,” Rev. Mod. Phys. 89(1), 015005 (2017). 10.1103/RevModPhys.89.015005

[13] Zhao T. Ourselin S. Vercauteren T. et al., “Seeing through multimode fibers with real-valued intensity transmission matrices,” Opt. Express 28(14), 20978–20991 (2020). 10.1364/OE.396734 32680147 PMC7470672

[14] Zhao T. Ourselin S. Vercauteren T. et al., “Focusing light through multimode fibres using a digital micromirror device: a comparison study of non-holographic approaches,” Opt. Express 29(10), 14269–14281 (2021). 10.1364/OE.420718 33985150 PMC8240458

[15] Zhao T. Ma M. T. Ourselin S. et al., “Video-rate dual-modal photoacoustic and fluorescence imaging through a multimode fibre towards forward-viewing endomicroscopy,” Photoacoustics 25, 100323 (2022). 10.1016/j.pacs.2021.100323 35028288 PMC8741494

[16] Zhao T. Pham T. T. Baker C. et al., “Ultrathin, high-speed, all-optical photoacoustic endomicroscopy probe for guiding minimally invasive surgery,” Biomed. Opt. Express 13(8), 4414–4428 (2022). 10.1364/BOE.463057 36032566 PMC9408236

[17] Zhao T. Ourselin S. Vercauteren T. et al., “High-speed photoacoustic-guided wavefront shaping for focusing light in scattering media,” Opt. Lett. 46(5), 1165 (2021). 10.1364/OL.412572 33649683 PMC8237830

[18] Borhani N. Kakkava E. Moser C. et al., “Learning to see through multimode fibers,” Optica 5(8), 960–966 (2018). 10.1364/OPTICA.5.000960

[19] Li Y. Yu Z. Chen Y. et al., “Image reconstruction using pre-trained autoencoder on multimode fiber imaging system,” IEEE Photonics Technol. Lett. 32(13), 779–782 (2020). 10.1109/LPT.2020.2992819

[20] Chen H. He Z. Zhang Z. et al., “Binary amplitude-only image reconstruction through a MMF based on an AE-SNN combined deep learning model,” Opt. Express 28(20), 30048–30062 (2020). 10.1364/OE.403316 33114890

[21] Meng L. Hu H. Hu J. et al., “Image reconstruction of multimode fiber scattering media based on deep learning,” Chin. J. Laser 47(12), 1206005 (2020). 10.3788/CJL202047.1206005

[22] Li D. Lin B. Wang X. et al., “High-performance polarization remote sensing with the modified U-Net based deep-learning network,” IEEE Trans. Geosci. Electron. 60, 5621110 (2022). 10.1109/TGRS.2022.3164917

[23] Caramazza P. Moran O. Murray-Smith R. et al., “Transmission of natural scene images through a multimode fiber,” Nat. Commun. 10(1), 2029 (2019). 10.1038/s41467-019-10057-8 31048712 PMC6497636

[24] Ju Z. Yu Z. Meng Z. et al., “Simultaneous illumination and imaging based on a single multimode fiber,” Opt. Express 30(9), 15596 (2022). 10.1364/OE.454850 35473276

[25] Changyan Z. Chan E. Wang Y. et al., “Image reconstruction through a multimode fiber with a simple neural network architecture,” Sci. Rep. 11(1), 896 (2020). 10.1038/s41598-020-79646-8 PMC7806887 33441671

[26] Han G. Hu H. Zhang Y. et al., “Seeing through multimode fibers with physics-assisted deep learning.,” Opt. Laser Technol. 167, 109761 (2023). 10.1016/j.optlastec.2023.109761

[27] Ronneberge O. Fischer P. Brox T., “U-Net: convolutional networks for biomedical image segmentation,” Medical Image Computing and Computer-Assisted Intervention 9351, 234–241 (2015). 10.1007/978-3-319-24574-4_28

[28] Adrian B. Tzimiropoulos G.. “Binarized convolutional landmark localizers for human pose estimation and face alignment with limited resources.” *ICCV*, (2017).

[29] Ozan O. Schlemper J. Folgoc L. L. et al., “Attention U-Net: learning where to look for the pancreas,” *CVPR*, (2018).

[30] Li D., “The MNIST database of handwritten digit images for machine learning research [best of the web],” IEEE Signal Process. Mag. 29(6), 141–142 (2012). 10.1109/MSP.2012.2211477

[31] Huang G. Liu Z. Maaten L. V. D. et al., “Densely connected convolutional networks,” In 2017 IEEE Conference on Computer Vision and Pattern Recognition (CVPR), 2261–2269.

[32] Wang F. Wang C. Deng C. et al., “Single-pixel imaging using physics enhanced deep learning,” Photonics Res. 10(1), 104 (2022). 10.1364/PRJ.440123

[33] Mitchell K. J. Turtaev S. Padgett M. J. et al., “High-speed spatial control of the intensity, phase and polarisation of vector beams using a digital micro-mirror device,” Opt. Express 24(25), 29269–29282 (2016). 10.1364/OE.24.029269 27958587

[34] Goorden S. A. Bertolotti J. Mosk A. P., “Superpixel-based spatial amplitude and phase modulation using a digital micromirror device,” Opt. Express 22(15), 17999–18009 (2014). 10.1364/OE.22.017999 25089419

[35] Zhong W. Dong Z. Pang C. et al., “Single multimode fibre for in vivo light-field-encoded endoscopic imaging,” Nat. Photonics 17, 1–9 (2022). 10.1038/s41566-023-01240-x

[36] Ye Z., “Speckle-image-reconstruction” 1.0, Github, (2025), https://github.com/Zye3/Speckle-Image-Reconstruction

